# Diseases of the Respiratory System

**Published:** 1904-10-08

**Authors:** 


### DISEASES OF THE RESPIRATORY SYSTEM.

(*Continued from page* 10.)

**Serum Treatment.**—The current hypothesis that Koch's tuberculin is a toxin, has been traversed by Marmorek[1] for the following reasons:—The same dose instead of producing uniform results when injected into a diseased animal, reacts very variably. In healthy animals no reaction is produced, but if ever so slight an infection with the tubercle bacilli is present an intense reaction is set up, although in cases riddled with tuberculosis the effect of a similar dose of tuberculin may be *nil*. Marmorek argues that to explain this and other anomalies in the action of tuberculin we must discard the idea that it is a toxin responsible for the lesions known as tuberculosis, but consider it as a product which stimulates the tubercle bacilli, and causes them to pour out an increased amount of the true toxin. Thus in slight lesions a reaction is produced, but when the lesions are extensive and large quantities of the true toxin are already being formed the extra stimulation makes very little difference, and no reaction is observed. Its curative value depends on an active immunisation caused by the true toxin, which is produced by the stimulation of the bacilli by Koch's tuberculin. As it is impossible to regulate this reaction, its effects must often be much in excess of what is desirable, and the animal is merely made worse by an excessive dose of the true toxin which results. Marmorek asserts that young tubercle bacilli (*primatifs*) differ from the fully developed organism, both in their morphology and staining properties, and also in producing no toxin on ordinary media. By growing them on a medium composed of leucotoxic serum and glycerinated liver bouillon, he contrived to keep their primitive character; though no tuberculin was produced, another toxic substance was found in the filtered cultures, which was regarded as the true tuberculo-toxin. With this substance Marmorek was able to immunise animals, and their serum, injected into other animals, was found to have a protective and curative action, produced by the process of "passive immunisation." Having satisfied himself that the injection of the serum thus produced was harmless, Marmorek proceeded to apply it to the cure of the disease in the human subject.[2] He observed that about 20 per cent. of cases, as shown by autopsy, recover spontaneously, and taking all methods of treatment together about the same percentage are cured. This does not mean that the chances of recovery for any individual are the same, whether he is treated in any particular manner or not, because we cannot say at the outset whether he is one of those very resistent persons who will recover naturally, or whether he is on the borderland and will only recover with whatever assistance it is possible to give him. What can be said is that he is not of the absolutely non resistent type, and it is this type alone which really decides whether a treatment is efficacious against the cause of the disease or not. Marmorek, therefore, began by treating apparently hopeless cases—with meningitis, advanced pulmonary lesions, etc.—and then gradually went down the scale until he arrived at a point at which his serum appeared to have a curative action. As a result of his observations he concluded that the important point was not the extent but the duration of the lesions—the most recent cases, even though acute and advancing, being most favourably influenced. With regard to meningitis, he considers that the negative results obtained are due to the impossibility of quite early diagnosis, and to some firm union of the toxin with the cells in the central nervous system, such as takes place in tetanus. Marmorek claims, however, to have improved some cases of meningitis, as regards severity of the symptoms, and with regard to phthisis to have cured cases of two years' standing or so, in which other treatment had failed. The extremely advanced cases, however, gave negative results. Hectic fever and dyspnœa (both toxic conditions) were much influenced, the effect on the expectoration and number of bacilli was variable, while hæmoptysis for an unexplained cause was checked. The results in "Surgical tuberculosis" and disease in the bones and joints were very encouraging. Marmorek insists that large doses continued over a long period are essential, and that minor unpleasant effects, though occasionally observed, are not more frequent or important than those seen with other sera. In England the serum has been tried on a not very extensive scale by Latham,[3] 30 severe cases having been treated over a period of three months. In all, 450 injections were given, and by careful aseptic precautions accidental infection was avoided. While pointing out the difficulties experienced in the selection of cases and in drawing deductions after so short a trial Latham concludes that the serum has a definite antitoxic effect, five cases being detailed in support of this view. In "surgical" cases and in the less severe forms of phthisis the symptoms appear to have improved, but severe pulmonary cases pursued their course uninfluenced. Various symptoms occurred attributable to the serum *per se*, and not to the antitoxin, such as pyrexia, rashes, coryza, neuralgic pains, etc., and in two or three cases more or less collapse. Many of these effects can be avoided by giving small doses and carefully watching the temperature, etc. In chronic cases 5 c.c. should be the maximum, and, after four daily injections, a period of three days' rest should be given. After three such series a fortnight's rest is advisable. In acute and "surgical" cases in adults doses of 8 to 10 c.c. are usually well borne, and in one child with meningitis 30 c.c. were injected for four days without ill effects. Another serum which is not yet on the market has been prepared by Béraneck,[4] a bacteriologist of Neuchâtel, and experimentally tried by Wilson in this country. It consists of a mixture of two substances—Basitoxin (BT) obtained by filtering a culture of the bacillus in a special medium, and Acidotoxin (AT) which is extracted by orthophosphoric acid from the bodies of the bacilli themselves. The mixture is said to be only slightly toxic and to act by increasing phagocytosis, and by rendering the tissues unfavourable to the growth of the bacillus. The injections must be continued for a considerable time. In lesions of the larynx, skin, bladder, joints, etc., local applications of the serum may be used. Sixty out of 90 cases treated during two or three years improved. Severe cases were, however, practically uninfluenced, though in early cases the cure was rapid.

[1] Lancet, Dec. 12, 1903, p. 1642. [2] Ibid, Mar. 26, 1904, p. 854. [3] Ibid, April 9, 1904, p. 979. [4] Brit. Med. Jour., Mar. 26, 1904, p. 735.

*(To be concluded.)*

